# Isolation and Host Range of Bacteriophage with Lytic Activity against Methicillin-Resistant *Staphylococcus aureus* and Potential Use as a Fomite Decontaminant

**DOI:** 10.1371/journal.pone.0131714

**Published:** 2015-07-01

**Authors:** Kyle C. Jensen, Bryan B. Hair, Trevor M. Wienclaw, Mark H. Murdock, Jacob B. Hatch, Aaron T. Trent, Tyler D. White, Kyler J. Haskell, Bradford K. Berges

**Affiliations:** Department of Microbiology and Molecular Biology, Brigham Young University, Provo, Utah, 84602, United States of America; ContraFect Corporation, UNITED STATES

## Abstract

*Staphylococcus aureus *(SA) is a commensal bacterium and opportunistic pathogen commonly associated with humans and is capable of causing serious disease and death including sepsis, pneumonia, and meningitis. Methicillin-resistant SA (MRSA) isolates are typically resistant to many available antibiotics with the common exception of vancomycin. The presence of vancomycin resistance in some SA isolates combined with the current heavy use of vancomycin to treat MRSA infections indicates that MRSA may achieve broad resistance to vancomycin in the near future. New MRSA treatments are clearly needed. Bacteriophages (phages) are viruses that infect bacteria, commonly resulting in death of the host bacterial cell. Phage therapy entails the use of phage to treat or prevent bacterial infections. In this study, 12 phages were isolated that can replicate in human SA and/or MRSA isolates as a potential way to control these infections. 5 phage were discovered through mitomycin C induction of prophage and 7 others as extracellular viruses. Primary SA strains were also isolated from environmental sources to be used as tools for phage discovery and isolation as well as to examine the target cell host range of the phage isolates by spot testing. Primary isolates were tested for susceptibility to oxacillin in order to determine which were MRSA. Experiments were performed to assess the host range and killing potential of newly discovered phage, and significant reductions in bacterial load were detected. We explored the utility of some phage to decontaminate fomites (glass and cloth) and found a significant reduction in colony forming units of MRSA following phage treatment, including tests of a phage cocktail against a cocktail of MRSA isolates. Our findings suggest that phage treatment can be used as an effective tool to decontaminate human MRSA from both hard surfaces and fabrics.

## Introduction

*Staphylococcus aureus* (SA) infections are the most frequent type of hospital-acquired infections reported in developed countries [1]. SA is a common commensal bacterium capable of colonizing the nose and skin and is found transiently in ~50% of the human population and ~20% permanently [2, 3]. Nasal colonization has been linked to surgical site infections [4] and SA can cause life-threatening diseases in many different tissue types including bones, joints, blood, lungs, heart, and brain [5]. SA is the bacterium most commonly associated with bloodstream, soft tissue, lung and skin infections [6]. Many of these infections are treated using antibiotics; however, bacterial evolution has resulted in strains of SA resistant to multiple antibiotics.

Methicillin Resistant *Staphylococcus aureus* (MRSA) represents a group of SA isolates commonly resistant to methicillin as well as erythromycin, levofloxacin, tetracycline, clindamycin, mupirocin, gentamicin, trimethoprim, and/or doxycycline but is typically susceptible to vancomycin [7]. Serious MRSA infections are increasingly difficult to treat using current antibiotics [8]. While MRSA infections rates have recently trended downwards, community acquired MRSA infections are now more common requiring treatment using antibiotics such as vancomycin [9, 10]. The concern is that vancomycin resistance seen in other bacteria (including some SA isolates) may be acquired by MRSA, thus leaving clinicians without any viable treatment options [11]. In April 2014 Rossi et al. highlighted this problem when they reported a case of MRSA resistant to vancomycin in Brazil [12]. There is a valid concern that vancomycin-resistant MRSA could become predominant in the near future and such infections may be untreatable. In 2012, there were an estimated 75,309 cases of invasive MRSA with 9,670 resulting in deaths within the United States [13]. A 2011 study estimated that the case fatality rate of invasive MRSA in the United States was about 25% [9] and another showed that SA bacteremia in New York City had a 30% mortality rate from 2002–2007 [14]. Researchers seeking new treatments for antibiotic-resistant bacteria have increasingly begun to look towards bacteriophage as a viable option in treating these infections, either in tandem with or as a replacement for antibiotics [15, 16].

Bacteriophages (phages) are viruses capable of infection and replication in bacterial cells. Phages are the most common organism found on the planet and as such represent great diversity in their overall host range [17, 18]. Since virus infectivity requires binding to a specific receptor, phage are specific for a small host range and are thus unable to infect human cells. Thus, the side effects associated with phage therapy of eukaryotic hosts are thought to be minimal [19]. The idea of using phage as a potential therapeutic tool has been around for as long as phage have been known to exist [19, 20] though some eastern European countries continued using phage as medical treatments and in some countries physicians still regularly practice phage therapy [21, 22]. Phage were used in the early 1900s to treat bacterial infections, but phage treatment was largely abandoned in favor of antibiotics in the 1940s [23]. Although bacteria can evolve to escape from phage-mediated killing, the use of a biological agent such as phage allows for evolution to also work in favor of phage re-acquiring the ability to lyse target cells [15, 24, 25]. Thus, it is thought that phage therapy could be superior to antibiotic therapy in terms of the ability of the treatment to evolve in response to the development of resistance by the target bacterium. Off-target effects of antibiotic therapy can have detrimental effects on non-pathogenic normal flora, but such effects are expected to be minimal with phage therapy [19].

In this report, we describe the isolation of 12 phages with lytic activity towards human SA/MRSA isolates. Virulent and temperate phages were found, isolated and purified using MRSA strains as hosts. We analyzed the lytic host range and lytic ability of each phage using spot tests and lytic culture assays of a panel of SA and MRSA cultures isolated from various human, livestock and environmental locations. In order to demonstrate the efficacy of our phage for clearing MRSA, we used our new phage to decontaminate MRSA from fomites and found a significant reduction in MRSA load from both a glass surface as well as fabric, which could be associated with nosocomial transmission. Further, we found significant reduction of MRSA loads when mixtures of MRSA isolates were treated with either single phage or with phage cocktails. Our results suggest that phage can be used as an effective way to decontaminate materials contaminated with MRSA.

## Materials and Methods

### Isolation of *Staphylococcus aureus* strains

Bacterial strains were isolated on Mannitol Salt agar (MSA) plates (Fluka Analytical). Plaque assays and spot tests for host range were performed on Luria-Bertani (LB) agar (10g tryptone, 5g yeast extract, 5g NaCl, 1mL 2N NaOH, 12g/L agar). LB broth contained 10g tryptone, 5g yeast extract, 5g NaCl, and 1mL 2N NaOH per liter. Top LB agar contained 4g/L agar, supplemented with 4mM MgCl_2_ and 4mM CaCl_2_. Phage buffer was made with 100mM NaCl, 10mM MgCl_2_, 50mM Tris-HCl, and 0.01% gelatin (pH 7.5).

Some strains were acquired from the American Type Culture Collection (Manassas, VA, USA) and others from BEI Resources (Manassas, VA). Additional strains of *S*. *aureus* were obtained from athletic facilities, hospitals, nasal swabs, environmental sampling, and from collaborators. To select for *S*. *aureus*, each sample was suspended in LB broth and thoroughly vortexed. 10μL of each sample was spotted onto a MSA plate, streaked to isolation and incubated at 37°C for 48h. Gram-staining, catalase and coagulase tube tests were performed on single colonies isolated on MSA plates to confirm *S*. *aureus* (gram positive cocci positive for catalase, coagulase and mannitol fermentation). Methicillin resistance was determined by plating on MSA plates with 2 μg/mL oxacillin, followed by incubation at 37°C for 48h (note that oxacillin resistance is considered to be equivalent to methicillin resistance in many studies; [7, 26]).

### Isolation of bacteriophage

#### Virulent phage isolation

Samples were obtained from the environment and stored at 4°C until phage enrichment. LB broth was added to dry samples and thoroughly vortexed to dislodge phage particles. An equal volume of 2x LB broth was added to liquid samples. All samples were then filtered using 0.45μm filters to remove bacteria. The filtrate was then added to a 4h culture of five different strains of SA: M1, M5, SA 29213, DH1 and HA1 (see Table 1). Samples were incubated overnight at 37°C shaking at 60rpm for phage enrichment. The overnight culture was centrifuged for 12min at 5,000xg to pellet bacteria, then filtered using a 0.45μm filter. 100μL of phage sample was added to 100μL of bacteria and incubated overnight at 37°C before adding to LB top agar for plaque production. Three rounds of successive plaque purifications were performed to isolate each phage. The bacterial strain used to isolate each phage is indicated in Table 2.

**Table 1:** SA/MRSA isolates used in these studies.

Strain ID	Source	Oxacillin
M1-9	Sports Training Center	Resistant
SA 29213, 6538, 4651	Purchased from ATCC	Susceptible
MRSA 43300	Purchased from ATCC	Resistant
USA300 LAC	Collaborator	Resistant
USA300 strains 0114, CA-127, CO-34, GA-92, NY-336, JE2	Acquired from BEI	Resistant
USA400 MW2	Collaborator	Resistant
USA400 HFH-30364	Acquired from BEI	Resistant
NS 6, 15	Nasal Swabs	Resistant
NS 13–14, 16, 22–23	Nasal Swabs	Susceptible
HA 1–5	Hospital	Resistant
DH 1–2	Dog Hair	Resistant
DH 3	Dog Hair	Susceptible
CJ 11	Raw Chicken	Susceptible
CJ 9	Raw Chicken	Resistant
RB 1	Raw Beef	Susceptible
TK 11	Raw Turkey	Resistant
TK 9	Raw Turkey	Susceptible

Isolates were confirmed as SA or MRSA as detailed in Methods. Methicillin resistance was tested by growth on MSA plates in the presence of 2μg/mL oxacillin. ATCC = American Type Culture Collection, NS = human nasal swab, HA = hospital acquired, DH = dog hair, CJ = raw chicken, RB = raw beef, TK = raw turkey.

**Table 2:** Phage isolates discovered in these studies.

Strain ID	Source	Isolating Strain
M1M5	Temperate/MRSA 5	M1
M1M4	Temperate/MRSA 4	M1
M1NS15	Temperate/NS 15	M1
M5NS22	Temperate/NS 22	M5
M5NS6	Temperate/NS 6	M5
SEW	Virulent/Sewage Influent	M1
CJ11	Virulent/Raw Chicken	M1
CJ12	Virulent/Raw Chicken	M1
CJ16	Virulent/Raw Chicken	DH1
CJ17	Virulent/Raw Chicken	DH1
CJ18	Virulent/Raw Chicken	DH1
CF6	Virulent/Chicken Feces	DH1

Temperate phages have a bipartite name where the first part reveals the bacterial strain used for isolation and the second part indicates the host strain. NS = human nasal swab, SEW = sewage influent, DH = dog hair, CJ = raw chicken, CF = chicken feces.

#### Temperate phage isolation

Log-phase SA or MRSA sub-cultures were grown for 30min at 37°C at 200rpm, followed by exposure to mitomycin C (Sigma-Aldrich) at 0.5μg/mL for 8h at 37°C. Cells were pelleted by centrifugation and supernatants passed through a 0.45μ filter and stored at 4°C until plaque assays were performed, as above.

High titer phage lysates were prepared by adding host bacteria and phage into LB top agar and overlaying onto LB agar plates. Overlays with near complete lysis, or a webbed plaque distribution, were treated with 4mL of phage buffer and the top agar overlay was crushed, followed by 90min incubation at room temperature. Phage buffer was removed and centrifuged at 5,000rpm for 10min and filtered at 0.45μm to remove bacterial cells, then stored at 4°C with chloroform. Phage stocks were titered by limiting dilution, using a similar protocol as used for plaque purification above.

### Host range analysis

#### Spot testing

Overnight cultures were prepared in LB medium and then sub-cultured by addition of 100μL to 3mL LB broth and grown at 37°C for 90min. 100μL of sub-culture was inoculated into 3mL of molten LB top agar and overlaid onto LB agar plates. Each overlay was allowed to solidify for 15min. All phage lysates (original titers approximately 10^8^pfu/mL) were diluted in 10 fold increments and 10μL of each dilution was spotted onto the bacterial overlay [27], dried, then incubated at 37°C overnight. As a control, each bacterial strain was also mock infected with sterile phage buffer. Results were analyzed based on detection of any lysis and further dilutions were checked for single plaques to ensure phage lysis rather than bacteriocin induced lysis. All spot tests were repeated in triplicate to confirm results.

#### Spectrophotometric assay of phage-treated liquid cultures

Overnight bacterial samples were sub-cultured in LB medium supplemented with 5mM CaCl_2_ and MgCl_2_ until reaching an OD_600_ of 1.4 by shaking at 200rpm at 37°C. At that point, 20μL of bacteria was inoculated into 3.5mL LB medium. Phage samples were diluted to 10^8^ pfu/mL in phage buffer. Bacterial samples were treated with 100μL of phage (or mock-treated with sterile phage buffer alone) into 3.5mL of culture. Samples were removed at 2, 3, and 4h after infection and the OD_600_ was measured on an Ultraspec 10 spectrophotometer (Amersham Biosciences, Piscataway, NJ, USA) using growth medium as a blank. Experiments were run in triplicate.

### Decontamination assays

#### Glass coverslip decontamination

Decontamination assays were performed to assess the ability of phage to clear MRSA from surfaces. 22 x 22 mm glass coverslips were used to test decontamination on hard surfaces. Coverslips were autoclaved and allowed to cool. 10μL of sterile milk was spread onto the surface and allowed dry, giving the coverslips a better surface for bacterial adherence [28]. The test MRSA strain was sub-cultured for 4h at 37°C to achieve logarithmic growth and then diluted 1:10^3^, giving a final concentration of approximately 10^6^ cfu/mL. 10μ of the culture was spread onto the coverslips and allowed to dry at room temperature (approx. 30min). 100μL of phage lysate was then added to the surface at a multiplicity of infection of 10 and incubated at room temperature for 30min. A mock treatment was performed using sterile phage buffer in place of phage lysate. To remove remaining bacteria from the surface, each coverslip was placed in a sterile 50mL conical tube containing 500μL of LB medium and vortexed at high-speed for 10s. Serial dilutions of each test were then plated on LB agar and incubated overnight at 37°C. Colonies were counted to determine bacterial load, and the ability of each phage to decontaminate the surface was calculated by dividing the mock-treated bacterial loads by the phage-treated bacterial loads.

#### Cloth decontamination

To test the utility of our phage for clearing SA/MRSA from items associated with nosocomial transmission, we tested lab coat material which was composed of 35% cotton and 65% polyester. 1.5 x 1.5cm pieces were prepared and autoclaved to achieve sterility. MRSA samples were sub-cultured for 4h to achieve logarithmic growth, then diluted 1:10^4^ and 100μL was added to the lab coat sample and allowed to remain for 30min at 37°C. Our baseline bacterial load recovered from untreated cloth was ~1-5x10^4^ cfu. 100μL of phage lysate was then added and incubated at 37°C for 30min. Phage titers added to the cloth ranged from 1x10^7^ to 1x10^8^ pfu for a range of multiplicity of infection of 200 to 50,000. As a control, sterile phage buffer alone was added as a mock treatment. Bacteria were removed by placing the cloth into 500μL LB medium followed by vortexing at high speed for 10s. The resulting medium was serially diluted, then plated onto an LB agar plate and incubated at 37°C overnight; colonies were counted the next morning. To determine the decontamination capability of the phage, colony-forming units were calculated and mock-treated bacterial loads were divided by phage-treated bacterial loads. These assays were performed in triplicate.

### Statistical analysis

**Fig 1:**
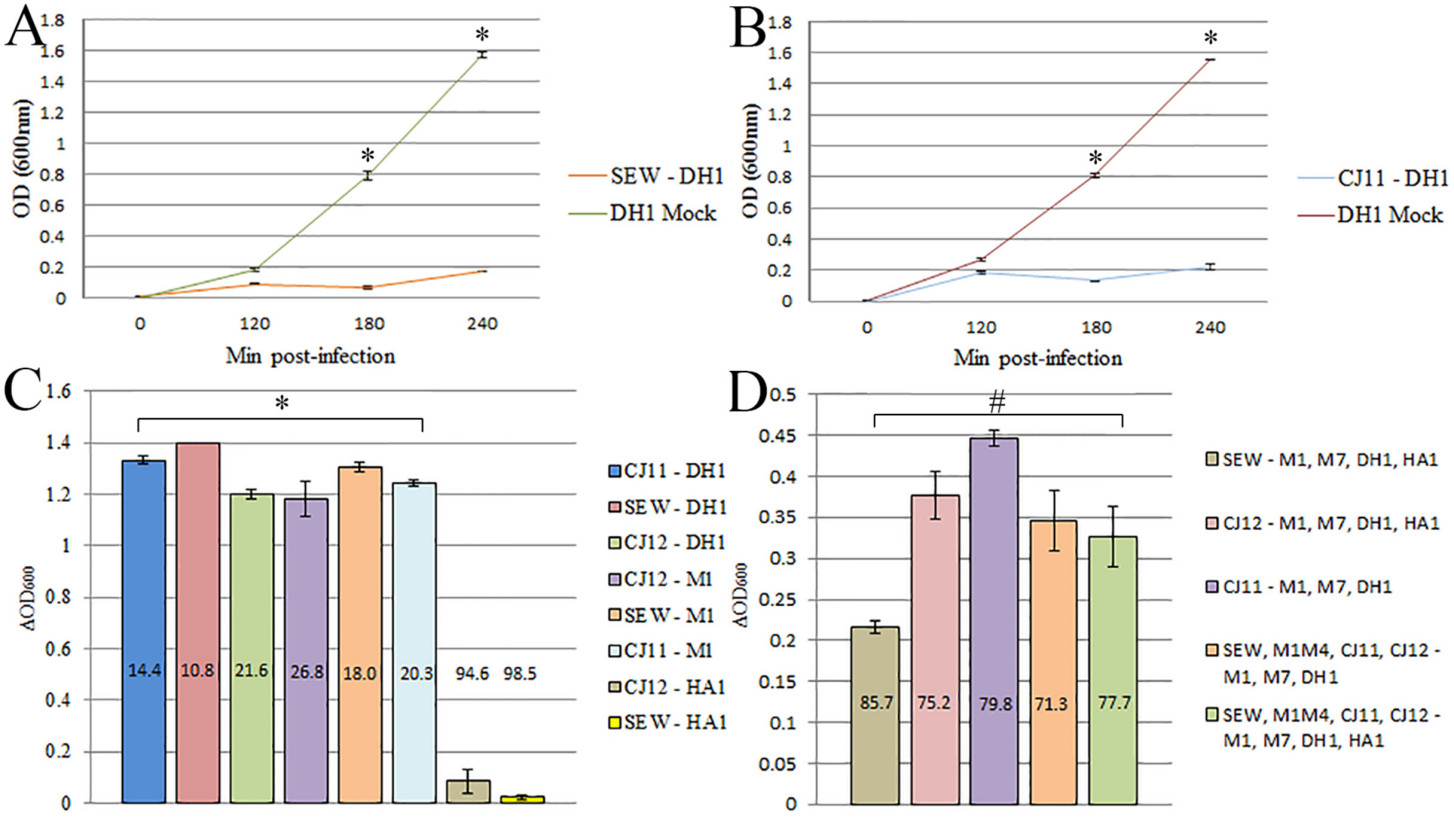
Assessment of host range by lysis of bacterial cultures. Phage were added to log phase bacterial cultures to assess host range and to determine the killing efficiency of the phages. Optical density was measured at 600nm to quantify cell density of the culture. A) MRSA strain DH1 infected with phage strain SEW across a time course. B) MRSA strain DH1 infected with phage strain CJ11 across a time course. C) A variety of bacterial strains were challenged with different phage and OD_600_ readings were taken at 4h. Results are reported in ΔOD_600_ units which were calculated as the difference between the OD_600_ of mock-treated cultures (sterile phage buffer only) and phage-treated cultures. D) Various combinations of bacterial strains were treated with either single phage strains or combinations phage. In panels C and D, the percent difference in OD_600_ readings (phage-treated divided by mock-treated) is also shown. All experiments were performed in triplicate; standard error is indicated. * p ≤ 0.002 by student’s t test. # p ≤ 0.01 by student’s t test when evaluating phage treated vs mock treated samples.

**Fig 2:**
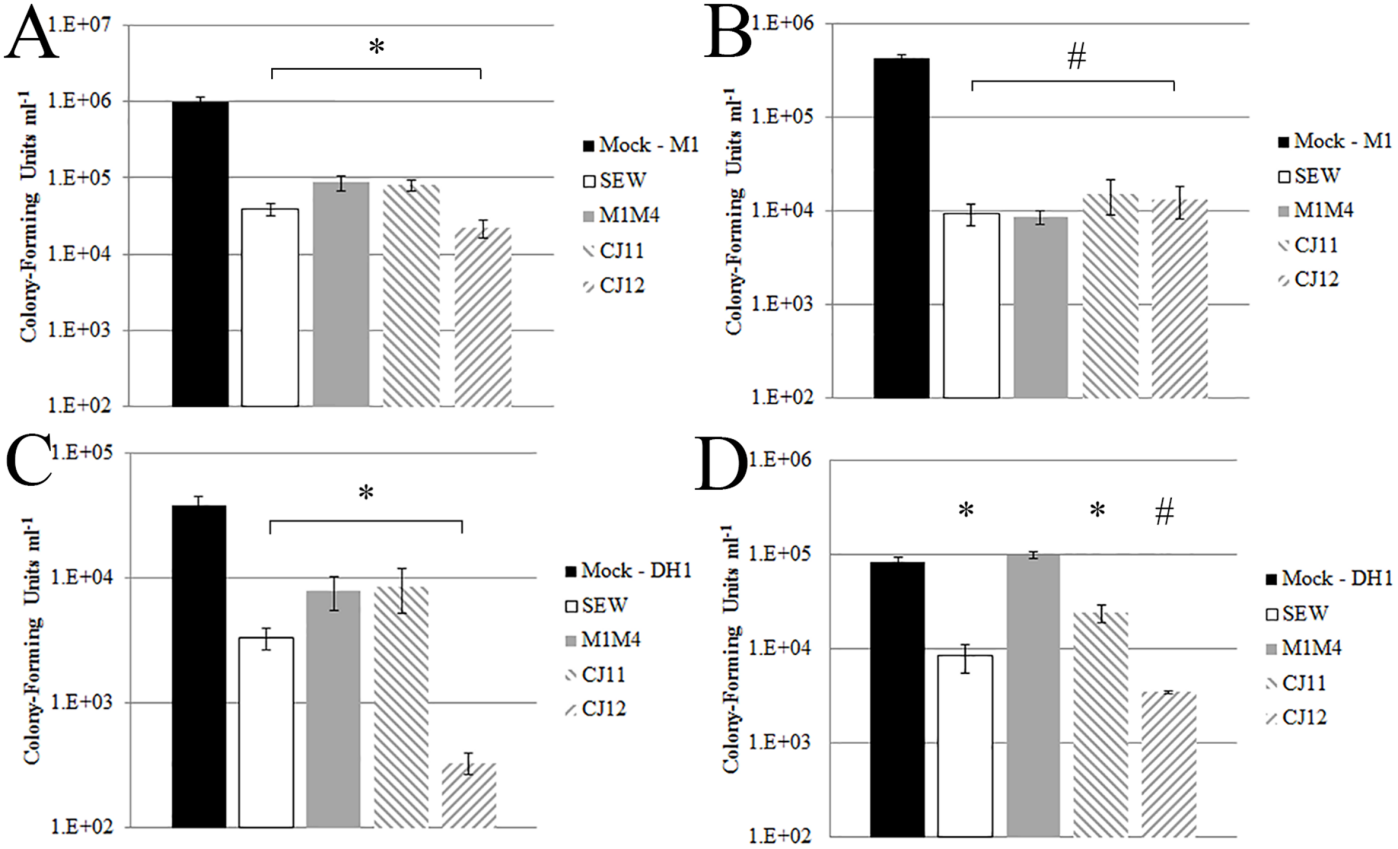
Decontamination of lab coats and glass coverslips. MRSA samples were inoculated onto either lab coat fabric or glass coverslips and then exposed to either a single phage or a mock phage treatment (sterile phage buffer only). The MOI ranged from 50 to 20,000. Bacteria were recovered and viable bacterial counts were determined by serial dilution and growth on LB agar plates. Results are reported as colony-forming units ml^-1^ A) MRSA strain M1 treated with either SEW, M1M4, CJ11 or CJ12 recovered from lab coat fabric. B) MRSA strain M1 treated with either SEW, M1M4, CJ11 or CJ12 recovered from glass coverslips. C) MRSA strain DH1 treated with either SEW, M1M4, CJ11 or CJ12 recovered from lab coat fabric. D) MRSA strain DH1 treated with either SEW, M1M4, CJ11, or CJ12 recovered from glass coverslips. Assays were performed in triplicate; standard error is indicated. * p ≤ 0.05 by unpaired, one-tailed student’s t test when evaluating phage-treated vs mock-treated samples. # p ≤ 0.005 by same student’s t test.

**Fig 3:**
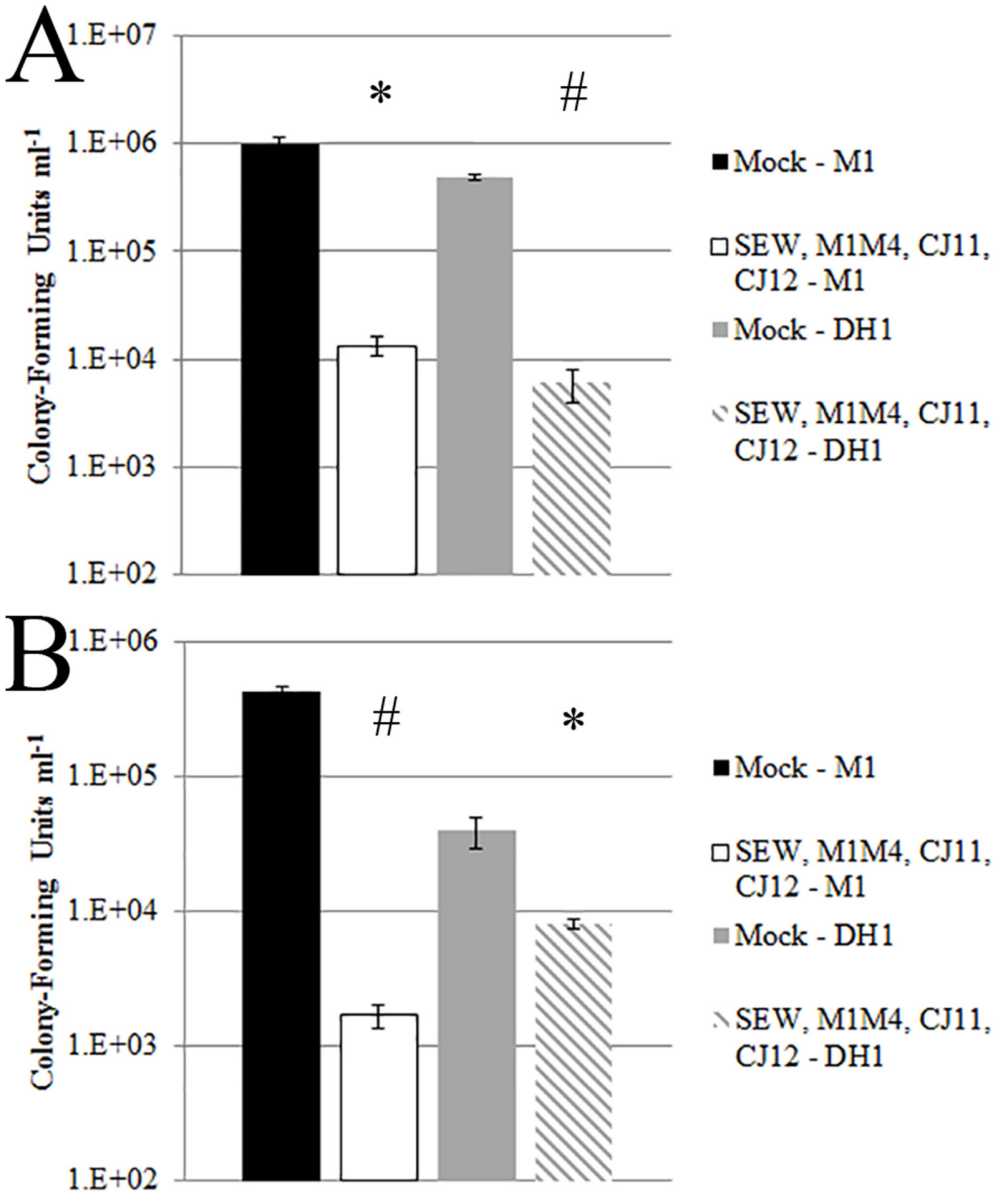
Decontamination of lab coats and glass coverslips with a phage cocktail. MRSA samples were inoculated onto either lab coat fabric or glass coverslips and then exposed to a phage cocktail consisting of SEW, M1M4, CJ11, CJ12, or mock phage treatment (sterile phage buffer only). The combined MOI ranged from 300 to 1,300. Bacteria were recovered from treated materials and viable bacterial counts were determined by serial dilution and growth on LB agar plates. A) MRSA strain M1 and DH1 treated with phage cocktail or mock treatment recovered from lab coat fabric. B) MRSA strain M1 and DH1 treated with phage cocktail or mock treatment recovered from glass coverslips. Assays were performed in triplicate; standard error is indicated. * p ≤ 0.05 by unpaired, one-tailed student’s t test when evaluating phage-treated vs mock-treated samples. # p ≤ 0.005 by same student’s t test.

Unpaired, one-tailed student’s t tests were performed in experiments from Figs 1, 2, and 3 in order to determine if significant differences existed between phage-treated and mock-treated samples. A value of p ≤ 0.05 was considered to be statistically significant.

### Ethics Statement

Brigham Young University Institutional Review Board (IRB) approval was granted for this study (protocol X14403). Informed written consent was obtained for each participant, and minors were not included in this study. Other human samples were provided by collaborators without any patient identifiers and were classified as exempt by the IRB.

## Results

### Isolation of *S*. *aureus* (SA) and methicillin-resistant *S*. *aureus* (MRSA) strains

A diverse group of SA and MRSA isolates were acquired during the course of these experiments in order to be used as tools for phage discovery and characterization. Some isolates were purchased from ATCC, some were acquired from collaborators, and others were isolated from the environment including from human, animal, and environmental sources. The following criteria were used to confirm isolation of SA or MRSA: growth on mannitol salt agar plates, ability to ferment mannitol, positive coagulase and catalase tests, and gram stains to confirm gram-positive cocci. Growth in the presence of 2 μg/mL oxacillin confirmed that some isolates were MRSA. A summary of the SA and MRSA isolates used in these studies is found in Table 1. In total, 42 SA strains were used including 30 MRSA and 12 methicillin-susceptible strains. 34 isolates were from human sources and 8 were from animal sources.

### Isolation of SA and/or MRSA-specific bacteriophage

Phages were isolated as described in methods, either from environmental samples or via induction of prophages. In total we isolated 12 SA-specific phages, all of which were isolated on and have lytic activity against MRSA strains (see Table 2). 5 of these were temperate phages induced from human SA isolates by mitomycin C treatment. The remaining 7 were virulent phages isolated from environmental samples. These samples were first suspended in LB broth and then filtered to remove bacteria. This filtrate was then incubated with a mixture of 5 SA/MRSA strains to amplify any phage present. 6 of the 7 virulent phage originated from poultry samples with the 7th originating from sewage influent. All phages were purified through three rounds of plaque purification, with the SA strain used listed in Table 2. Following plaque purification, high titer phage stocks were produced and titered by limiting dilution.

### Assessment of host range of phage isolates

One goal of this study was to determine the specificity of phage for particular SA/MRSA isolates, with the expectation that some phage would have a broader tropism than others due to presence/absence of phage receptor molecules or intracellular restriction mechanisms. To assess the tropism of the phage described in Table 2, spot tests were performed on plates containing lawns of various SA/MRSA isolates as described in Methods. Plates were assessed for the lytic ability of each phage isolate by monitoring for clearing of bacterial lawns (Table 3) and all spot tests were repeated to confirm the validity of the results. All bacterial isolates shown in Table 1 were tested for host range, although many isolates were not lysed by any of the phages and so those results are not shown in Table 3. Based upon spot testing results, at least 6 unique phages were isolated: CJ12, SEW, M1M4, CJ17, a cluster including M5NS6 and M5NS22, and a cluster including M1M5, CJ18, CJ11, CF6, M1NS15, and CJ16. Interestingly, some members of the latter cluster (M1M5 and M1NS15) were found via mitomycin C induction of prophage while the others were found as virulent phages (CJ18, CJ11, CF6, CJ16).

**Table 3:** Assessment of host range by spot testing.

Strain ID	Virulent phages							Temperate phages				
	SEW	CJ12	CJ16	CJ11	CJ18	CF6	CJ17	M1M4	M1M5	M1NS15	M5NS6	M5NS22
M1	X	X	X	X	X	X		X	X	X		
M5								X			X	X
M7	X	X	X	X	X	X	X	X	X	X		
DH1	X	X	X	X	X	X	X	X	X	X		
NS13	X							X				
HA1	X	X						X				
HA3											X	X

Following plaque purification, phages were tested for host range by spot testing on lawns of SA and MRSA isolates. Detection of any lawn clearing is indicated by an X. All testing was performed in triplicate, and virus stocks were serially diluted to confirm presence of phage lysis (discreet plaque formation) rather than bacteriocin activity.

Phage M1M4 had the broadest host range, with lytic ability against 6 different bacterial strains. CJ17 and the M5NS6 cluster had the narrowest host range, with only 2 different bacterial strains lysed. Of the isolated phages, all had activity against human MRSA isolates, possibly because a cocktail of human MRSA isolates were used to enrich phage in the early steps of this study. We also noted that 3 bacterial strains (M1, M7, DH1) were lysed by most of the new isolated phages, possibly due to the lack of prophage in these isolates. The majority of the bacterial isolates examined were not lysed by any of the phages. One possible explanation for this finding is that half of the phages (6 of 12) were isolated from an animal source while only 8 of the 42 SA strains tested came from animal sources.

In order to confirm the host range results, and to determine the relative lytic ability of the various phages, liquid bacterial cultures were exposed to phage and spectrophotometric assays were performed in log phase cultures. Since some bacterial isolates produce toxins, it is possible that phage stocks contained such toxins which could lead to false positive results on a spot test. Such toxins are expected to be too dilute to be effective in the large volumes used for a spectrophotometric assay but could be active in the spot test procedure, thus the spot tests were diluted until single plaques were visible. We found that changes in the optical density of bacterial cultures were routinely noted at 180 and 240min post-infection (Fig 1A and 1B). In subsequent experiments, readings were only taken at the 240min time point. We calculated the difference in OD_600_ readings between mock-treated and phage-treated cultures for a variety of phage and bacterial strain combinations and results are reported in ΔOD_600_ units in Fig 1C and 1D. We found significant reductions in OD_600_ readings for many different combinations of phage and bacterial targets, including significant differences when one phage was targeted to multiple bacterial isolates or if multiple phages were used to target multiple bacterial isolates (Fig 1D).

### Assessment of phage ability to decontaminate fomites

Nosocomial transmission of SA/MRSA is a major problem, especially when the immunocompromised and those with underlying health issues become infected. We sought to determine if our phages could effectively decontaminate fomites associated with nosocomial transmission. We used the results of the spot tests to design combinations where the phages were predicted to have a host range that would include the bacterial targets. Decontamination of fabric was also analyzed as a more likely source of nosocomial transmission. We used glass coverslips to represent decontamination of solid surfaces.

To imitate a contaminated fomite we inoculated a single MRSA strain onto sterile cloth (from a lab coat similar to one worn by clinicians) and then added a single phage and determined the bacterial load following either phage treatment or mock treatment. Multiple phage attachment incubation time points were tested to find the ideal time point for target cell lysis (5min, 10 min, 15 min, and 30 min) and results are presented in S1 Fig. The only significant results detected were that a 30 min. phage cocktail treatment showed a significant reduction in CFUs in comparison to the 10 min. phage treatment on lab coat fabric, and the 10 min. phage treatment showed a significant reduction in CFU in comparison to the 30 min. phage treatment on glass coverslips. Subsequent experiments were performed at 30 min. Mock treatments were performed using sterile phage buffer alone.

We found significant reductions in the numbers of MRSA colony forming units (CFUs) in tests where one phage/one MRSA was used to decontaminate lab coat fabric (Fig 2). Decontamination of MRSA strain M1 inoculated fabric with one of four phages yielded a 1–1.5 log reduction of CFUs ml^-1^ compared to mock treated samples (Fig 2A). Each different phage treatment had a p-value ≤ 0.01. MRSA DH1 was similarly inoculated and treated with phages (Fig 2C) and resulted in 0.5–1 log reductions in CFUs in each test with a p-value ≤ 0.05.

We inoculated sterile glass coverslips with MRSA (see Methods) and then treated with singular phages or mock treatments and measured the ability to decontaminate bacteria (Fig 2B and 2D). Glass slides inoculated with MRSA strain M1 and treated with singular phage showed highly significant reductions in CFUs (p ≤ 0.005) compared to mock treatments (Fig 2B). Each phage treatment yielded at least a 1.5 log reduction in CFUs with two phages nearly achieving a 2 log reduction. Coverslips inoculated with MRSA strain DH1 also showed significant decreases in CFUs as compared to the mock treatment (Fig 2D), with the exception of M1M4 treatment. CJ11 treated coverslips achieved a 0.5 log reduction (p ≤ 0.02), while SEW and CJ12 achieved a 1 log (p ≤ 0.01) and 1.5 log reduction (p ≤ 0.007), respectively.

We combined phages M1, M1M4, CJ11, and CJ12 to create a phage cocktail to measure potential synergistic ability to decontaminate MRSA from lab coat material or glass coverslips (Fig 3). The phage cocktail was designed to include the most efficiently lytic phages as well as those with the broadest tropism. Using the phage cocktail we found significant reductions in CFUs when decontaminating either lab coat fabric (Fig 3A) or glass coverslips (Fig 3B). In treating the lab coat fabric we observed a nearly 2 log reduction in CFUs for both M1 and DH1 strains. In studies using MRSA strain DH1, we found a highly significant decrease in CFUs on fabric with a p value ≤ 0.008 (Fig 3A), similar to the phage cocktail treatment of MRSA strain M1 on a glass coverslip with a p value ≤ 0.003 (Fig 3B).

## Discussion

In this study, we isolated 12 new bacteriophages with lytic activity against SA and MRSA. We determined the host range of these phages by both spot testing and spectrophotometric assays of phage-treated bacterial cultures. We then examined the ability of single phages or phage cocktails to decontaminate MRSA from a glass surface as well as from fabric. We found that our phages were able to significantly reduce MRSA growth in culture, and that they were able to significantly reduce colony-forming units of human MRSAs from both glass and fabric.

The host range tests carried out in these studies showed that our newly isolated phage tended to have greater lytic activity against human SA strains as compared to non-human isolates, and to also lyse MRSA more commonly than methicillin-susceptible SA. Most of our virulent phage isolates were found in sources related to chickens. Phage isolated from chicken sources would not necessarily be predicted to have activity against MRSA or human SA isolates. However, these findings may be related to the protocol used to initially enrich phage, wherein 5 SA strains were used: 4/5 were MRSA and 4/5 were human SA isolates. Other phage may have been present during enrichment, but we selected for those with lytic activity against human MRSA in our enrichment protocol.

Measurement of the optical densities of phage-treated MRSA cultures revealed that very efficient lysis occurred. The density of phage-treated cultures after 4h was essentially the same as 2h. After comparing the results of the spot tests and spectrophotometric assays, we found that the results were mostly in agreement with one another. Some anomalies were seen in this comparison, such as when spot tests indicated lytic ability for a given phage/bacterial strain but culture assays failed to show significant lysis. Two examples are shown in Fig 1C (e.g., SEW and CJ12 treatment of isolate HA1). When we tested more challenging scenarios for phage reduction of bacterial samples (e.g., one phage targeted to 3–4 bacterial strains in Fig 1D) we still detected significant reductions in bacterial cell density. The cell density was higher in such experiments, despite the fact that spot testing predicted that all bacterial strains could be lysed. We partially attributed these findings to MRSA strain HA1, which was not lysed efficiently in culture assays. When strain HA1 was removed from one such experiment, the cell density dropped (Fig 1D and data not shown) indicating that HA1 was less susceptible to clearing in a culture assay.

We opted to use glass as a model test surface for MRSA decontamination, as has been tested previously by others [28]. Significant reductions in bacterial CFUs were detected in nearly all experiments when using either glass or fabric. One experiment failed to show a significant reduction for phage strain M1M4 (see Fig 1D), but this strain was discovered as a temperate phage and so the lytic potential is expected to be less than that seen for virulent phages. Since M1M4 had the broadest host range of our phages, we still included this phage in subsequent testing. Many decontamination experiments showed a reduction in CFUs greater than 1 log, and occasionally 2 log reductions were seen. Cocktails of phage were typically more effective at decontaminating MRSA than single phage.

We conclude that we have isolated at least 6 unique phage with lytic activity against human MRSA isolates. These phage have robust activity in both liquid culture and in decontaminating hard surfaces and fabrics associated with nosocomial transmission. Our future plans include further characterization of these phage by genome sequencing and transmission electron microscopy, and further testing for decontamination potential under varying circumstances.

## Supporting Information

S1 Fig Time course decontamination of lab coats and glass coverslips. A decontamination time course assay was done to identify the optimal time to expose the bacterial to phage before recovering cells. MRSA samples were inoculated onto either lab coat fabric or glass coverslips and then exposed for 5 min., 10 min., 15 min. or 30 min. to either a phage cocktail or a mock phage treatment (sterile phage buffer only; results not shown). The MOI ranged from 50 to 20,000. Bacteria were recovered and viable bacterial counts were determined by serial dilution and growth on LB agar plates. Results are reported as colony-forming units ml^-1^ A) MRSA strain DH1 inoculated onto lab coat fabric and then treated with a phage cocktail of SEW, M1M4, CJ11 and CJ12. The 30 min. phage treatment showed a significant reduction in CFUs in comparison to the 10 min. phage treatment. B) MRSA strain DH1 inoculated onto glass coverslips and then treated with a phage cocktail of SEW, M1M4, CJ11 and CJ12. The 10 min. phage treatment showed a significant reduction in CFU in comparison to the 30 min. phage treatment. Assays were performed in triplicate; standard error is indicated. * p < 0.05 by unpaired, one-tailed student’s t test when evaluating the various time points relative to each other.
(TIF)
