# Supplementary figures and images for: Isolation and Host Range of Bacteriophage with Lytic Activity against Methicillin-Resistant Staphylococcus aureus and Potential Use as a Fomite Decontaminant
Source: PLoS One. 2015 Jul 1;10(7):e0131714. doi: 10.1371/journal.pone.0131714 (PMC4488860; doi:10.1371/journal.pone.0131714)

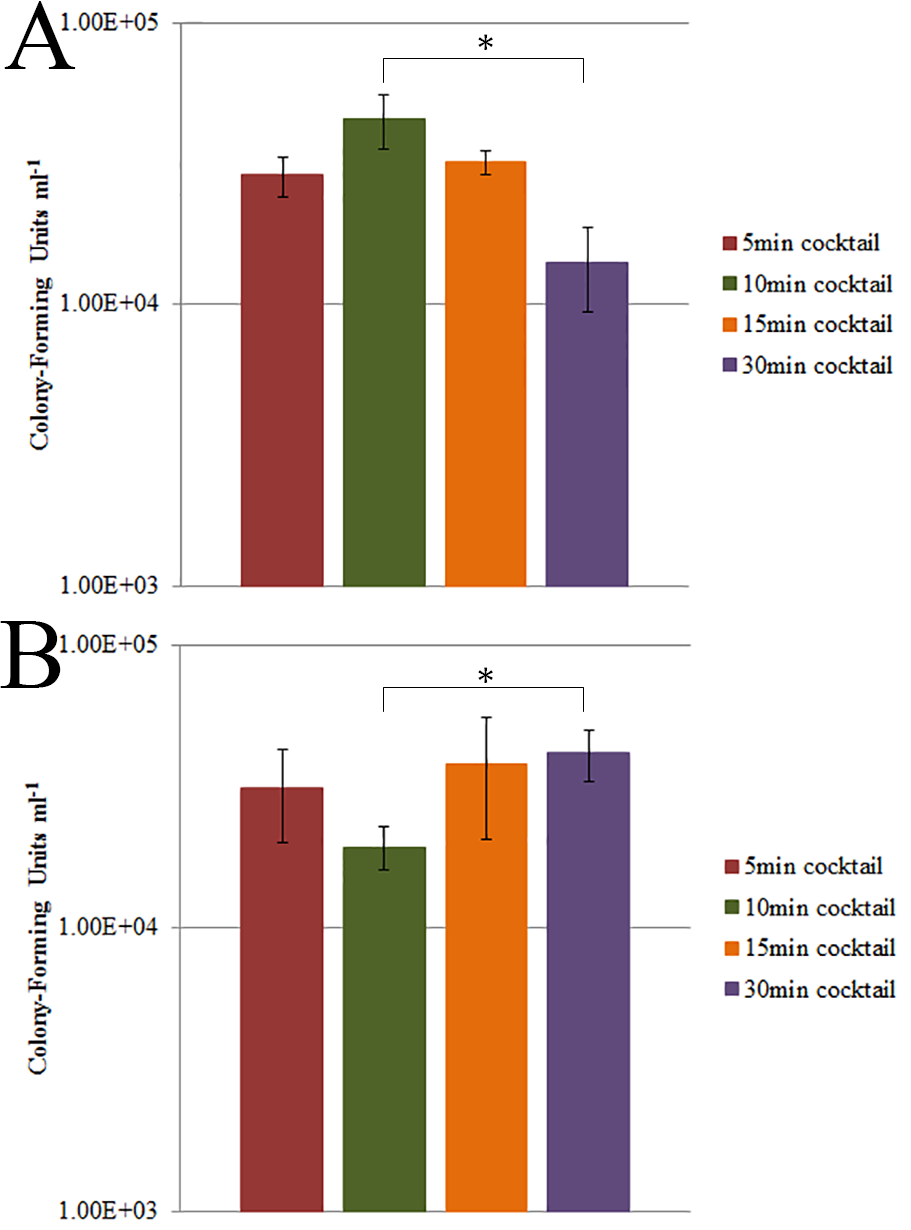

Supplement: S1 Fig — A decontamination time course assay was done to identify the optimal time to expose the bacterial to phage before recovering cells. MRSA samples were inoculated onto either lab coat fabric or glass coverslips and then exposed for 5 min., 10 min., 15 min. or 30 min. to either a phage cocktail or a mock phage treatment (sterile phage buffer only; results not shown). The MOI ranged from 50 to 20,000. Bacteria were recovered and viable bacterial counts were determined by serial dilution and growth on LB agar plates. Results are reported as colony-forming units ml-1 A) MRSA strain DH1 inoculated onto lab coat fabric and then treated with a phage cocktail of SEW, M1M4, CJ11 and CJ12. The 30 min. phage treatment showed a significant reduction in CFUs in comparison to the 10 min. phage treatment. B) MRSA strain DH1 inoculated onto glass coverslips and then treated with a phage cocktail of SEW, M1M4, CJ11 and CJ12. The 10 min. phage treatment showed a significant reduction in CFU in comparison to the 30 min. phage treatment. Assays were performed in triplicate; standard error is indicated. * p < 0.05 by unpaired, one-tailed student’s t test when evaluating the various time points relative to each other. (TIF) [file pone.0131714.s001.tif]
